# Discharge locations after hospitalizations involving opioid use disorder among medicare beneficiaries

**DOI:** 10.1186/s13722-022-00338-x

**Published:** 2022-10-08

**Authors:** Patience Moyo, Melissa Eliot, Asghar Shah, Kimberly Goodyear, Eric Jutkowitz, Kali Thomas, Andrew R. Zullo

**Affiliations:** 1 Department of Health Services, Policy, and Practice, Brown University School of Public Health, 121 South Main Street, Box G-S121-6, Providence, RI 02912 USA; 2 Center for Gerontology and Healthcare Research, Brown University School of Public Health, Providence, RI USA; 3 Department of Epidemiology, Brown University School of Public Health, Providence, RI USA; 4 Brown University, Providence, RI USA; 5 Department of Psychiatry and Human Behavior, Brown University, Providence, RI USA; 6 Center for Alcohol and Addiction Studies, Department of Behavioral and Social Sciences, Brown University School of Public Health, Providence, RI USA; 7 Providence VA Medical Center, Center of Innovation in Long Term Services and Supports, Providence, RI USA

**Keywords:** Opioid use disorder, Opioid-related disorders, Hospitalization, Post-acute care, Medicare

## Abstract

**Background:**

Hospitalizations involving opioid use disorder (OUD) have been increasing among Medicare beneficiaries of all ages. With rising OUD-related acute care use comes the need to understand where post-acute care is provided and the capacities for OUD treatment in those settings. Our objective was to describe hospitalized Medicare beneficiaries with OUD, their post-acute care locations, and all-cause mortality and readmissions stratified by post-acute care location.

**Methods:**

We conducted a retrospective cohort study of acute hospitalizations using 2016–2018 Medicare Provider Analysis and Review (MedPAR) files linked to Medicare enrollment data and the Residential History File (RHF) for 100% of Medicare fee-for-service beneficiaries. The RHF which provides a person-level chronological history of health service utilization and locations of care was used to identify hospital discharge locations. We used ICD-10 codes for opioid dependence or “abuse” to identify OUD diagnoses from the MedPAR file. We conducted logistic regression to identify factors associated with discharge to an institutional setting versus home adjusting for demographics, comorbidities, and hospital stay characteristics.

**Results:**

Our analysis included 459,763 hospitalized patients with OUD. Of these, patients aged < 65 years and those dually enrolled in Medicaid comprised the majority (59.1%). OUD and opioid overdose were primary diagnoses in 14.3% and 6.2% of analyzed hospitalizations, respectively. We found that 70.3% of hospitalized patients with OUD were discharged home, 15.8% to a skilled nursing facility (SNF), 9.6% to a non-SNF institutional facility, 2.5% home with home health services, and 1.8% died in-hospital. Within 30 days of hospital discharge, rates of readmissions and mortality were 29.7% and 3.9%; respectively, with wide variation across post-acute locations. Factors associated with greater odds of discharge to institutional settings were older age, female sex, non-Hispanic White race and ethnicity, dual enrollment, longer hospital stay, more comorbidities, intensive care use, surgery, and primary diagnoses including opioid or other drug overdoses, fractures, and septicemia.

**Conclusions:**

More than one-quarter (25.8%) of hospitalized Medicare beneficiaries with OUD received post-acute care in a setting other than home. High rates and wide variation in all-cause readmissions and mortality within 30 days post-discharge emphasize the need for improved post-acute care for people with OUD.

**Supplementary Information:**

The online version contains supplementary material available at 10.1186/s13722-022-00338-x.

## Background

There is growing evidence of rising rates of opioid use disorder (OUD) and opioid-related hospitalizations and emergency department (ED) among older adults and younger adults with long-term disabilities, many of whom are enrolled in Medicare [1–7]. Medicare beneficiaries represent an increasing proportion of people with OUD in the United States. Medicare is also the primary payer in roughly 40% of all OUD-related inpatient stays nationally [8]. Nevertheless, important knowledge gaps remain about health service use and outcomes among Medicare beneficiaries diagnosed with OUD. The prevailing patterns of increasing opioid-related acute care use and overdose deaths emphasize the need to better understand populations whose growing risk of opioid-related harms may be overlooked. This inattention may be due in part to societal perceptions about who is at risk and suboptimal recognition and screening for OUD in population subgroups [9]. Older adults in particular are often perceived to be at low risk of OUD and other substance use disorders despite growing evidence suggesting otherwise [4, 6, 7, 9, 10].

Several other circumstances magnify the urgency to better understand and respond to rising OUD diagnoses and related acute care use in the Medicare population [11, 12]. First, Medicare beneficiaries have a high prevalence of chronic, painful, and disabling conditions [13–15] and are commonly prescribed opioids that may contribute to the development or exacerbation of OUD [16]. Second, high rates of polypharmacy and resulting drug interactions may compound the risk of serious adverse events such as opioid overdose among those receiving chronic opioid therapy [17]. Third, older and disabled Medicare beneficiaries have significant multimorbidity, including concurrent respiratory, cardiovascular, and renal conditions, that may make them less likely to survive an overdose [18–20]. Lastly, much is unknown about post-acute care use following OUD-related hospitalizations and regarding the extent to which diverse settings of post-acute care are equipped to provide or facilitate access to OUD treatment.

Post-acute care plays an important role in the healthcare system and involves rehabilitation, recuperation, symptom management, and continued medical treatment after an individual has undergone acute hospital care. Varied post-acute care options exist, including skilled nursing facilities (SNFs), inpatient rehabilitation facilities, long-term care hospitals, and home health agencies [21, 22]. Institutional post-acute care is often needed for medically complex patients with multiple chronic conditions, functional limitations, serious infections, and other complications, who are unable to immediately return home safely after hospital discharge. The extent of institutional post-acute care use by people with OUD is unknown and deserves attention especially as regulatory and logistical issues may create unique challenges to prescribing and administering medications to treat OUD (MOUD). Due to federal regulations, SNFs which are a dominant setting for institutional post-acute care, cannot administer methadone and must coordinate with an opioid treatment program (OTP) to transport the patient to the OTP or deliver methadone to the facility. To provide buprenorphine, facilities require an X-waivered prescriber or relationships with external prescribers. The 2021 practice guideline changes for administering buprenorphine for OUD, which exempted certain practitioners from X-waiver training requirements, may serve as an opportunity to enhance care continuity and access to guideline-recommended care for OUD, including MOUD, in post-acute facilities.

Our objective was to characterize Medicare beneficiaries with OUD-related acute hospitalizations, describe their post-acute care locations, and describe their post-hospitalization outcomes stratified by post-acute care location. We hypothesized that receipt of post-acute care in institutional settings is common among hospitalized patients with OUD and that older adults represent a higher proportion of beneficiaries who are discharged to locations other than home. Examining Medicare beneficiary characteristics and their settings of care after OUD-related hospitalizations can inform understanding of the post-acute care needs of this population. Developing this understanding can promote the integration of addiction treatment with both geriatric/primary care and post-acute medical care in order to holistically address the behavioral health and other medical needs of patients across the care continuum [23].

## Methods

### Data

We used the national Medicare Provider Analysis and Review (MedPAR) file for 2016–2018 linked to the Medicare Master Beneficiary Summary File (MBSF) and the Residential History File (RHF) [24]. Medical diagnoses and characteristics of inpatient stays were identified from MedPAR data. The MBSF contains information about beneficiary enrollment (e.g., Medicare Advantage participation and dual Medicare/Medicaid coverage) and demographic characteristics. The RHF is a data algorithm that provides a person-level daily chronological history of health service use and location of care per calendar year by linking enrollment, claims, and assessment data [24]. We used the RHF to determine the hospital discharge location and to identify readmissions and mortality.

### Study sample

Our study population included adult (18 years of age and older) Medicare beneficiaries residing in the 50 states and Washington D.C. who had acute inpatient stays involving OUD. We excluded beneficiaries enrolled in Medicare Advantage as they may not generate complete claims. See Additional file 1: Appendix S1 for the sample selection diagram. We identified OUD using ICD-10 codes for opioid ‘abuse’ or dependence captured in any diagnosis position on inpatient claims [25]. We refer to these as OUD-related hospitalizations. All eligible OUD-related hospitalizations were included meaning that individuals could contribute multiple hospitalizations.

### Measures

Among patients who were discharged alive, the location of post-acute care was captured within one day of the hospital discharge date. The hospital discharge location was classified into four mutually exclusive categories namely community without home health services, community with home health services, SNFs—a nursing home or distinct part of a nursing home, and non-SNF institutional/inpatient setting (i.e., long-term care hospitals, inpatient rehabilitation facilities, psychiatric units). In addition to clinical considerations of the level of care across different post-acute care settings, our categorization was informed by the distribution of the data and consideration of SNFs as a distinct location that has been the focus of the emerging literature on referral and admission practices for hospitalized patients with OUD [26–30]. As such, we chose to specify SNFs as a standalone location because they are a dominant setting for post-acute care and to advance the state of knowledge specific to SNF use and outcomes among people with OUD. We grouped together the other institutional settings to facilitate readability and avoid small cell sizes.

Transfers to another acute care hospital were considered as a single hospitalization. Due to the data structure of the RHF, the subsequent location for individuals who chose to leave the hospital before the treating physician recommended discharge (i.e., patient-directed discharges sometimes referred to as “leaving against medical advice”) was home without home health unless there were claims or assessments that indicated care in other locations or receipt of home health services. A variable for beneficiary discharge status in inpatient claims allowed estimation of patient-directed discharges; however, the accuracy of the measurement is unclear. It is possible that some beneficiaries were discharged directly to hospice; however, hospice claims are not location specific because hospice services can be provided in community or institutional settings. For this reason and because of the expected small numbers of referrals to hospice, we did not directly assess hospice as a hospital discharge location. We analyzed in-hospital death as an additional hospital discharge disposition.

To understand the clinical profiles of hospitalized Medicare beneficiaries with OUD, we assessed their primary diagnosis for the hospitalization and calculated a Gagne comorbidity score to summarize disease burden and estimate mortality risk [31, 32]. We report select primary diagnoses including OUD, opioid and other drug overdoses, septicemia, and common diagnoses for inpatient stays among Medicare beneficiaries. We measured the characteristics of hospital stays for patients with OUD based on the length of the hospital stay and intensive care unit (ICU) use. We measured the rates of all-cause mortality and any readmission during the 30 days following hospital discharge.

### Analysis

We conducted a descriptive analysis where we calculated frequencies, proportions, and means and standard deviations to characterize who has OUD-related hospitalizations and their location of care following hospital discharge. We further used multivariable logistic regression models to identify factors associated with discharge to an institutional setting compared with home (with or without home health services) adjusting for demographics, comorbidities, and hospital stay characteristics including length of stay, ICU use, receipt of surgical procedures, and primary diagnoses. Separate models were implemented for the overall sample and stratified by age under 65 vs. 65 and older. Regression analyses accounted for repeated measures at the beneficiary level. Data analysis was conducted using R version 4.0.1 (Vienna, Austria) and SAS version 9.4 (Cary, North Carolina) software. The Brown University Institutional Review Board designated the study as exempt.

## Results

### Primary diagnosis during hospitalization

**Fig. 1:**
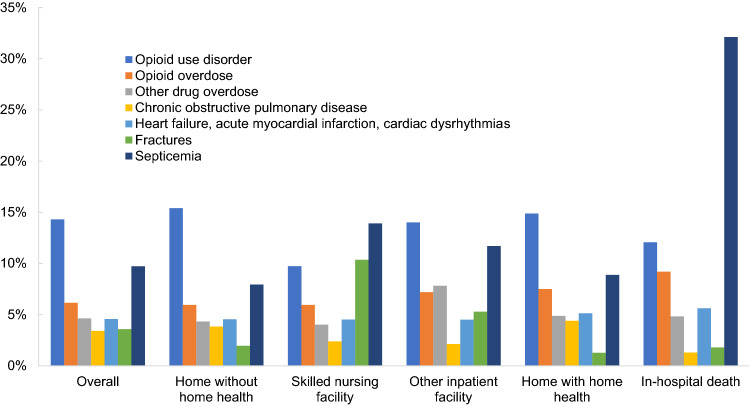
Prevalence of select primary diagnoses among fee-for-service Medicare beneficiaries with OUD-related hospitalizations during 2016–18. The indicated conditions were selected because they directly relate to drug-related harm (OUD and overdose) or are common reasons for hospitalizations in Medicare. Refer to Additional file 1: Appendix S2 to see the specific percentages

Our study included 459,763 acute inpatient stays with a primary (14.3%) or secondary (85.7%) diagnosis of OUD among Medicare fee-for-service beneficiaries during 2016–18. There were 8291 deaths during hospitalization. Septicemia was common among in-hospital decedents with almost one-third (32.1%) identified with this primary diagnosis (Fig. 1). Refer to Additional file 1: Appendix S2 for the actual percentages represented in Fig. 1. Almost 1 in 10 of in-hospital decedents had a primary diagnosis of opioid overdose. Among beneficiaries who survived a hospitalization, the share with a primary diagnosis of OUD was lowest in SNFs (9.7%) compared with other locations. A non-trivial proportion of beneficiaries across all post-acute care settings had an opioid (6.0% to 7.5%) or other drug overdose (4.0% to 7.8%) reported as the primary diagnosis during hospitalization. Overall, opioid and other drug overdoses were a primary diagnosis in 6.2% and 4.6% of OUD-related hospitalizations; respectively.

### Sample demographics and post-acute care location

Dual Medicare and Medicaid enrollment was common (59.1%) and older adults (at least 65 years of age) accounted for 39.7% of OUD-related hospitalizations in Medicare (Table 1). We found that 70.3% of OUD-related acute hospitalizations were discharged home, 15.8% to a SNF, 9.6% to a non-SNF institutional setting, 2.5% home with home health services, and 1.8% died in-hospital. Hospital discharge codes in inpatient claims estimated patient-directed discharges at 5.1% (data not shown). The distribution of the location of post-acute care varied for older adults compared with Medicare beneficiaries under 65 years of age. Beneficiaries discharged to SNFs tended to be older compared to those in other post-acute care locations or who died in the hospital. Two-thirds of OUD-related hospitalizations receiving post-acute care in SNFs involved older adults. Relative to their proportion in the overall sample (53.7%) of OUD-related hospitalizations, there was a higher share of beneficiaries of female sex who went to SNFs (60.2%) or received home health (57.1%). Non-Hispanic White beneficiaries were represented in higher proportions among those discharged to SNFs or non-SNF institutional settings; whereas, minorities accounted for a smaller share of beneficiaries discharged to institutional post-acute care settings relative to the overall sample.

**Table 1:** Demographic, comorbidity, and health care utilization characteristics of opioid use disorder-related hospitalizations among fee-for-service Medicare beneficiaries, 2016–18

Characteristic, %^a^	Overall	Hospital discharge location				
		Home without home health	Skilled nursing facility (SNF)	Non-SNF institutional setting*	Home with home health	In-hospital death
	N = 459,763	n = 323,307	n = 72,595	n = 44,077	n = 11,493	n = 8291
Discharge location, as percent of total stays^b^	100	70.3	15.8	9.6	2.5	1.8
Age, mean (SD), years	59.7 (14.6)	57.5 (14.3)	68.8 (12.4)	58.8 (14.6)	61.3 (13.3)	65.1 (13.7)
18–49	24.5	28.6	6.9	26.6	18.9	13.0
50–64	35.8	37.7	27.4	35.1	38.5	32.5
65–74	16.2	15.3	19.6	16.0	17.7	19.9
75 +	23.6	18.4	46.2	22.4	25.0	34.6
Female sex	53.7	52.5	60.2	51.8	57.1	49.9
Race and ethnicity						
Non-hispanic white	79.9	78.5	85.2	82.6	77.4	81.2
Non-hispanic black/African American	14.2	15.3	10.7	11.9	17.2	12.8
Hispanic	2.5	2.7	1.5	2.1	2.4	2.3
Other	3.4	3.5	2.7	3.4	3.0	3.7
Medicaid dual enrollment	59.1	59.7	56.6	59.8	63.9	49.5
Year of hospitalization						
2016	34.0	34.2	33.2	34.2	34.0	33.7
2017	34.0	33.9	34.1	35.0	31.4	34.1
2018	32.0	31.9	32.8	30.9	34.5	32.2
*Hospital stay attributes*						
Length of hospital stay, mean (SD), days	5.5 (6.6)	4.9 (5.7)	7.8 (8.2)	6.4 (7.9)	4.6 (5.4)	8.3 (11.1)
Length of hospital stay ≥ 3 days, %	71.3	66.9	93.4	70.4	60.5	70.0
Combined Comorbidity Score, mean (SD)	2.8 (2.1)	2.5 (2.0)	3.6 (2.2)	2.8 (2.1)	3.2 (2.2)	4.0 (2.2)
Comorbidity count ≥ 3, %	47.3	42.6	64.2	47.7	55.5	70.4
Intensive care unit use, %	31.3	27.5	37.0	42.2	29.1	73.0
*Post-hospital discharge outcomes within 30 days*						
All-cause acute readmission, %	29.7^c^	27.7	26.3	47.5	40.4	–
All-cause mortality, %	3.9^c^	3.1	5.7	6.4	4.7	–

^*^Non-SNF institutional settings include inpatient rehabilitation facilities, long term care hospitals, psychiatric hospitals

^a^Unless otherwise indicated

^b^Row percent; whereas, other percentages are column percents

^c^Denominator for percent is 451,472 excluding in-hospital deaths

### Post-acute care locations and outcomes in age subgroups

The mean age among younger Medicare beneficiaries was 50.5 (standard deviation: 10.4) years compared with 73.5 (standard deviation: 7.1) years among older adults. Among older adults, 59.5% were discharged home and 26.1% to SNFs; whereas, among beneficiaries under 65 years of age, 77.4% were discharged home and 9.0% to SNFs. In-hospital mortality was 2.5% among older adults versus 1.4% among beneficiaries under 65 years. See Additional file 1: Appendix tables S3 and S4. Patient-directed discharges were more prevalent among the under 65 subgroup (7.1%) compared with older adults (1.9%) (data not shown). Rates of 30-day all-cause readmissions were 26.1% (≥ 65 years) and 32.1% (< 65 years) and 6.2% (≥ 65 years) and 2.4% (< 65 years) for 30-day all-cause mortality.

### Multimorbidity, hospital stay attributes, and outcomes by hospital discharge disposition

We observed that beneficiaries with more comorbid conditions were more likely to die in-hospital or be discharged to SNFs (Table 1). Overall, almost one-third of OUD-related hospitalizations involved ICU use and the average length of hospitalization was almost 6 days. ICU use and length of inpatient stay were positively correlated with in-hospital mortality and receipt of post-acute care in an institutional setting. The overall prevalence of 30-day all-cause readmissions was 28.1% and 3.8% for 30-day all-cause mortality. Almost half (47.5%) of hospital discharges to ‘non-SNF institutional settings’ resulted in a readmission within 30 days. Readmissions were lowest among those discharged to SNFs (26.3%). All-cause 30-day mortality ranged from 3.1% (home without home health) to 6.4% (non-SNF institutional settings).

### Regression results

Overall, the odds of being discharged to an institutional post-acute care location increased with older age, female sex, non-Hispanic White race and ethnicity, dual Medicare and Medicaid enrollment, longer duration of hospitalization, greater number of comorbidities, ICU use, and receipt surgical procedures during hospitalization (Table 2). Beneficiaries whose primary diagnoses for hospitalization were opioid or other drug overdoses (OR = 1.93, 95% CL 1.87, 1.99), fractures (OR = 3.73, 95% CL 3.61, 3.86) or septicemia (OR = 1.30, 95% CL 1.27, 1.33) had greater odds of receiving post-acute care from an institutional setting rather than home. Having OUD as a primary diagnosis was associated with lower odds (OR = 0.57, 95% CL 0.55, 0.58) of post-acute care in institutional settings. The directions of the associations in age subgroups were the same as in the overall sample with the exception of males having greater odds of being discharged to institutional settings among beneficiaries under 65 years of age.

**Table 2:** Factors associated with discharge to institutional post-acute settings among hospitalized Medicare beneficiaries with OUD, overall and stratified by age group

Demographic characteristics	Odds ratio (95% Confidence Limits)		
	Overall	Age < 65 years	Age ≥ 65 years
Age			
< 50 vs. < 50–64	0.65 (0.63, 0.67)	0.67 (0.65, 0.68)	–
65–74 vs. 50–64	1.55 (1.52, 1.59)	–	–
75–84 vs. 50–64	2.28 (2.22, 2.33)	–	1.47 (1.44, 1.51)^a^
85 + vs. 50–64	3.77 (3.67, 3.89)	–	2.45 (2.37, 2.53)^a^
Male vs. female	0.95 (0.94, 0.97)	1.03 (1.01, 1.06)	0.88 (0.86, 0.90)
Race and ethnicity			
Black vs.White, Non-Hispanic	0.75 (0.73, 0.77)	0.79 (0.77, 0.82)	0.69 (0.67, 0.72)
Hispanic vs.White, Non-Hispanic	0.76 (0.72, 0.80)	0.80 (0.75, 0.86)	0.65 (0.58, 0.73)
Other vs.White, Non-Hispanic	0.85 (0.81, 0.89)	0.90 (0.85, 0.96)	0.79 (0.74, 0.85)
Dual Medicare and Medicaid enrollment vs. Medicare only	1.58 (1.55, 1.61)	1.45 (1.42, 1.49)	1.72 (1.68, 1.76)
Hospital stay characteristics			
Length of stay ≥ 3 vs. < 3 days	2.30 (2.26, 2.34)	1.67 (1.63, 1.71)	3.35 (3.26, 3.45)
Gagne comorbidity score ≥ 3 vs. < 3	1.35 (1.33, 1.37)	1.35 (1.32, 1.38)	1.33 (1,30, 1.36)
Intensive care unit use vs. none	1.36 (1.34, 1.38)	1.46 (1.43, 1.49)	1.23 (1.21, 1.26)
Surgical procedure vs. none	1.21 (1.19, 1.22)	1.21 (1.19, 1.24)	1.22 (1.19, 1.24)
Primary diagnosis			
Opioid use disorder	0.57 (0.55, 0.58)	0.54 (0.52, 0.57)	0.70 (0.67, 0.74)
Opioid or other drug overdose	1.93 (1.87, 1.99)	1.94 (1.86, 2.02)	1.56 (1.48, 1.64)
Cancer	0.72 (0.68, 0.77)	0.77 (0.70, 0.86)	0.68 (0.63, 0.73)
Chronic obstructive pulmonary disease	0.63 (0.60, 0.65)	0.64 (0.60, 0.69)	0.61 (0.57, 0.65)
Heart failure, acute myocardial infarction, cardiac dysrhythmias	0.77 (0.75, 0.80)	0.89 (0.84, 0.95)	0.71 (0.68, 0.74)
Fracture	3.73 (3.61, 3.86)	3.28 (3.11, 3.46)	3.94 (3.76, 4.12)
Septicemia	1.30 (1.27, 1.33)	1.47 (1.43, 1.52)	1.14 (1.10, 1.18)
Year of hospitalization			
2017 vs. 2016	1.01 (0.99, 1.02)	1.04 (1.02, 1.07)	0.98 (0.96, 1.00)
2018 vs. 2016	0.97 (0.95, 0.99)	0.99 (0.96, 1.01)	0.95 (0.93, 0.97)

^a^Reference category is 65–74 years

## Discussion

Rising rates of OUD and opioid overdoses contribute to increasing opioid-related hospitalizations and may increase the demand for post-acute care. This national study of all acute hospitalizations involving OUD among fee-for-service Medicare beneficiaries during 2016–18 provides insights about who experienced these hospitalizations, where they went following hospital discharge, and how they fared with respect to 30-day readmissions and mortality. Several results stood out in our analysis. First, OUD-related hospitalizations among our sample were characterized by high proportions of beneficiaries of female sex, Non-Hispanic White race and ethnicity, age under 65 years, or dually-enrolled in Medicare and Medicaid. Second, among approximately one-quarter of OUD-related hospitalizations, the hospital discharge location was an institutional setting such as a SNF, long-term care hospital or inpatient rehabilitation facility. Third, OUD-related hospitalizations among Medicare beneficiaries were resource intensive with one-third requiring ICU use and more than one-quarter resulting in all-cause readmission within 30 days post-hospitalization. Additionally, we found that OUD was predominantly a secondary, rather than a primary diagnosis among these hospitalizations; this observation suggests that this population often has co-occurring non-OUD diagnoses warranting inpatient care and emphasizes the importance of post-acute care that addresses behavioral health and other medical issues.

It is concerning that among hospitalized Medicare beneficiaries with OUD nearly 30% had a hospital readmission for any reason and almost 4% died within 30 days post-discharge. In contrast, an analysis of data from an academic medical center in Boston Massachusetts reported a prevalence of hospital readmissions among patients with OUD at 18.2% [33]. Furthermore, the readmission rates among people with OUD in our analysis were greater than in Medicare generally where readmissions were estimated at 16.9% in 2018 [34]. Within age subgroups, our finding that all-cause mortality rates were more than twice as high among older adults with OUD than among younger adults with OUD is consistent with prior research conducted among Veterans receiving care in the U.S. Veterans Health Administration [35]. Similar to prior research, we found that older adults with OUD had more chronic conditions than younger adults with OUD which could partially explain the observed differences in mortality rates. As such, integrating OUD-specific and standard geriatric care for older adults could improve health outcomes after hospitalization. Furthermore, the high burden of 30-day readmissions and mortality among all hospitalized Medicare beneficiaries with OUD emphasizes the need for improved post-hospital care for people who are discharged home as well as to post-acute care facilities.

The U.S is undergoing rapid expansion of the older adult population, which is projected to double in size from 2016 to 2060 [36, 37]. This demographic shift could be accompanied by a substantially higher prevalence of OUD and related outcomes as cohorts with more liberal attitudes toward opioid use enter older adulthood and become eligible for Medicare [37, 38]. In particular, the cohort born during 1946 to 1964 is thought to have experimented more with alcohol and illicit drugs than did previous generations [39, 40]. Opportunities exist for hospital practitioners to begin OUD treatment and facilitate the transition of care to the outpatient setting [41]. Simultaneously, it is essential to understand and respond to the needs of patients who could benefit from initiating or continuing treatment for OUD in post-acute care settings. Women, among whom opioid overdose deaths have increased at a disproportionately faster rate than for men and who accounted for majority of Medicare beneficiaries receiving institutional post-acute care in our analysis, may deserve particular consideration in efforts to increase access to OUD treatment [42].

National practice guidelines recommend the use of medications to treat OUD; however, questions exist about the appropriateness and feasibility of providing OUD treatment in different locations for post-acute and long-term care. Moreover, only 15.9% of beneficiaries with OUD received medication for OUD (MOUD) through Medicare in 2020 [43], adding to concerns that Medicare beneficiaries face substantial barriers accessing treatment [44]. Common reasons for not receiving treatment for substance use disorders among Medicare beneficiaries include financial barriers (insurance coverage and affordability), concerns about stigma, uncertainty about treatment effectiveness, and logistical barriers such as lack of transportation and not knowing where to get help from [45]. A systematic evaluation of access to MOUD in different post-acute care settings is lacking. The few existing studies predominantly focus on barriers to SNF post-acute care among hospitalized patients with OUD [26, 27], and there is hardly any empirical evidence to provide insight about the availability of MOUD in other post-acute care settings. Nonetheless, expert opinions indicate that barriers to MOUD access are widespread not only in SNFs [28, 29, 46], but also in other long-term care facilities as well as home health agencies. [47–49]

There are indications that SNFs are receiving more referrals for patients diagnosed with OUD and that many facilities deny admission due to the presence of substance use or MOUD despite this practice violating antidiscrimination laws [26, 30, 46]. A recent Massachusetts study based on electronic referrals to post-acute care facilities from a large medical center found that 81.8% of referrals were rejected and that use of MOUD was explicitly listed as reason for rejection in 37.4% of OUD hospitalizations [26]. People with OUD who gain admittance to SNFs may face policy and practice impediments to accessing MOUD given that federal law prohibits dispensing methadone for OUD outside maintenance clinics. Furthermore, the availability of providers who prescribe buprenorphine treatment for OUD in SNFs is unknown, but is likely scant because the lack of clinicians on staff who can prescribe buprenorphine has been suggested as a potential reason for SNFs denying admission to people with OUD [26].

Efforts to reduce or eliminate regulatory, reimbursement, and logistical barriers that hamper MOUD initiation or continuation for Medicare beneficiaries in institutional settings for post-acute and long-term care are needed. Given Drug Enforcement Agency (DEA) regulations that prohibit the dispensing of methadone outside highly-regulated clinics, considerations of the practicality of transporting individuals to methadone clinics or delivering MOUD to SNFs and other institutional settings must be considered. Stocking buprenorphine or naltrexone medications to treat OUD in post-acute or long-term care pharmacies could be another avenue to reduce barriers to accessing OUD treatment during post-acute and long-term care. Medicare reimbursement for MOUD also needs to be flexible to cover OUD treatment during post-acute and long-term stays outside the community. Such modifications stand to benefit not only older adults who are more likely to be discharged to institutional post-acute care locations but also younger adults who research indicates are entering SNFs in greater numbers due in part to housing instability [50, 51]. Additional investigation is needed to elucidate the extent to which social needs rather than (or combined with) the need rehabilitation or the other traditional services required post-hospitalization contribute to Medicare beneficiaries being discharged to SNFs.

## Study limitations

Our study had some limitations. First, outpatient medical and pharmacy claims data were unavailable; therefore, we lack information about the history of OUD diagnosis and receipt of MOUD. However, our study provides a complete national picture of all Medicare inpatient acute care with a diagnosis of OUD. Second, a limitation inherent to secondary analysis of administrative claims is potential underreporting or misclassification of OUD status owing to coding practices that may prioritize diagnoses with higher reimbursement [52, 53]. Prior reports indicate that OUD is typically underdiagnosed among older adults; therefore, the underreporting of OUD status may be greater among older Medicare beneficiaries compared with beneficiaries under 65 years of age [9]. Third, this analysis captures the ultimate post-discharge location as billed and thus lacks contextual information regarding clinical recommendations for discharge, patient and family preferences, or rejections of referrals to post-acute care facilities. Lastly, although the information provided by the RHF is comprehensive, patient-directed discharges cannot be determined using the RHF, it does not capture out-of-pocket expenses and mainly pertains to the Medicare fee-for-service population because Medicare Advantage beneficiaries may have incomplete claims due to capitated payments.

## Conclusions

Our findings demonstrated that more than one-quarter of OUD-related hospitalizations in Medicare resulted in discharge to institutional post-acute care settings. The high rates of 30-day all-cause readmissions and mortality further highlight the relevance and importance of improving post-acute care for hospitalized patients with OUD. Opportunities exist to not only raise awareness about the post-acute care needs of individuals with OUD but to also develop strategies that optimize the quality of care for people with OUD of all ages and in diverse locations. While federal legislation and major initiatives by the Centers for Medicare and Medicaid Services (CMS) promise to improve Medicare beneficiaries’ access to MOUD in community settings [54], the impact of these efforts in facilitating access to evidence-based care for OUD in institutional care settings is unclear and deserves consideration. As the U.S. faces substantial projected growth of the older adult population, it is critical to examine the appropriateness, capacity and willingness for institutional post-acute and long-term care settings to deliver care for OUD that is consistent with national practice guidelines.

## Supplementary Information

**Additional file 1: Appendix S1.** Sample selection flow diagram indicating hospital discharge location following OUD-related hospitalizations. **Appendix S2.** Percentages of select primary diagnoses identified during OUD-related hospitalizations in Medicare during 2016-2018. **Appendix S3.** Demographic, comorbidity and health care utilization characteristics of opioid use disorder-related hospitalizations among older adult (age ≥65 years) fee-for-service Medicare beneficiaries, 2016-18. **Appendix S4.** Demographic, comorbidity and health care utilization characteristics of opioid use disorder-related hospitalizations among younger (age <65 years) fee-for-service Medicare beneficiaries, 2016-18.

## Data Availability

The data used in this study is unavailable due to the constraints of data use agreements with the Centers for Medicare and Medicaid Services (CMS).

## Abbreviations

OUD: Opioid use disorder
MedPAR: Medicare Provider Analysis and Review
RHF: Residential History File
ICD-10: International Classification of Diseases Tenth Revision
SNF: Skilled nursing facility
ED: Emergency department
MOUD: Medication for opioid use disorder
DEA: Drug Enforcement Agency
