# Supplementary material for: Discharge locations after hospitalizations involving opioid use disorder among medicare beneficiaries
Source: Addict Sci Clin Pract. 2022 Oct 8;17:57. doi: 10.1186/s13722-022-00338-x (PMC9548174; doi:10.1186/s13722-022-00338-x)
Supplement: Supplementary file 1 — Additional file 1: Appendix S1. Sample selection flow diagram indicating hospital discharge location following OUD-related hospitalizations. Appendix S2. Percentages of select primary diagnoses identified during OUD-related hospitalizations in Medicare during 2016-2018. Appendix S3. Demographic, comorbidity and health care utilization characteristics of opioid use disorder-related hospitalizations among older adult (age ≥65 years) fee-for-service Medicare beneficiaries, 2016-18. Appendix S4. Demographic, comorbidity and health care utilization characteristics of opioid use disorder-related hospitalizations among younger (age <65 years) fee-for-service Medicare beneficiaries, 2016-18. [file 13722_2022_338_MOESM1_ESM.docx]

**Supplementary Material**

**Appendix S1**: Sample selection flow diagram indicating hospital discharge location following OUD-related hospitalizations


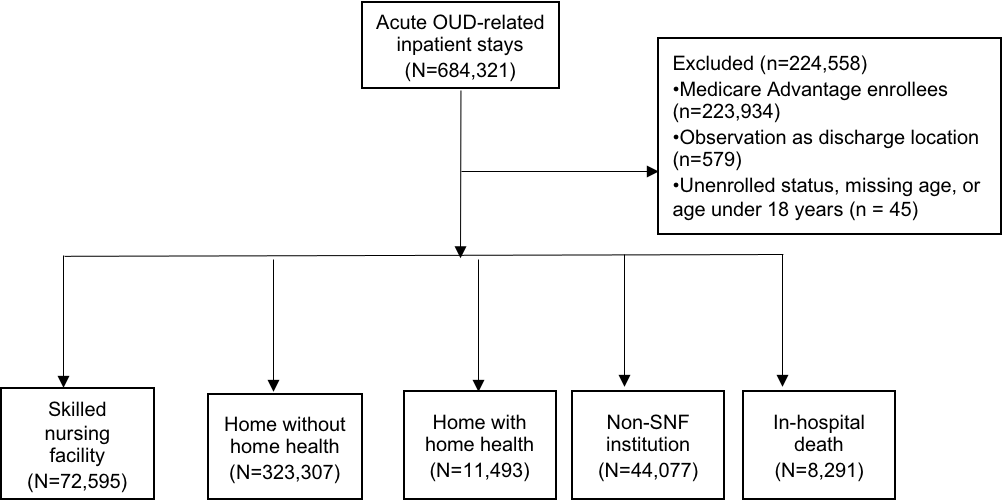


The analyzed sample totaled 459,763 observations. Skilled nursing facility (SNF) claims and Minimum Data Set (MDS) assessments identified from the Residential History File (RHF) were grouped under the SNF hospital discharge location. Home health use was determined using home health claims and Home Health Outcome and Information Assessment (OASIS) data from the RHF.

**Appendix S2**: Percentages of select primary diagnoses identified during OUD-related hospitalizations in Medicare during 2016-2018

|  |  | **Hospital discharge disposition** | | | | |
| --- | --- | --- | --- | --- | --- | --- |
| **Primary diagnosis** | Overall | Home without home health | Skilled nursing facility | Other inpatient facility* | Home with home health | In-hospital death |
|  | N=459,763 | N=323,307 | N=72,595 | N=44,077 | N=11.493 | N= 8,291 |
| Opioid use disorder | 14.3% | 15.4% | 9.7% | 14.0% | 14.9% | 12.1% |
| Opioid overdose | 6.2% | 5.9% | 6.0% | 7.2% | 7.5% | 9.2% |
| Non-opioid drug overdose | 4.6% | 4.3% | 4.0% | 7.8% | 4.9% | 4.8% |
| Septicemia | 9.7% | 7.9% | 13.9% | 11.7% | 8.9% | 32.1% |
| Chronic obstructive pulmonary disease | 3.4% | 3.8% | 2.4% | 2.1% | 4.4% | 1.3% |
| Heart failure, acute myocardial infarction, cardiac dysrhythmias | 4.6% | 4.5% | 4.5% | 4.5% | 5.1% | 5.6% |
| Fractures | 3.6% | 2.0% | 10.4% | 5.3% | 1.3% | 1.8% |
| Cancer | 1.5% | 1.4% | 1.4% | 1.1% | 1.3% | 4.2% |
| Diabetes | 1.7% | 1.6% | 1.9% | 1.5% | 2.7% | 0.5% |
| Renal disease | 1.4% | 1.4% | 1.4% | 1.0% | 2.0% | 1.5% |
| Osteoarthritis | 1.5% | 1.4% | 2.7% | 0.6% | 0.6% | -* |

* Suppressed due to small sample size

**Appendix S3**: Demographic, comorbidity and health care utilization characteristics of opioid use disorder-related hospitalizations among older adult (age ≥65 years) fee-for-service Medicare beneficiaries, 2016-18

|  |  | Hospital discharge location | | | | |
| --- | --- | --- | --- | --- | --- | --- |
| Characteristic, %^a^ | Overall | Home without home health | Skilled nursing facility (SNF) | Non-SNF institutional setting* | Home with home health | In-hospital death |
|  | N=182,889 | n=108,894 | n=47,696 | n=16,888 | n=4,894 | n= 4,517 |
| **Discharge location,** **as percent of total stays**^b^ | 100 | 59.5 | 26.1 | 9.2 | 2.7 | 2.5 |
| **Age, mean (SD), years** | 73.5 (7.1) | 72.6 (6.6) | 75.7 (7.8) | 73.2 (6.8) | 73.1 (6.7) | 74.8 (7.9) |
| 65-74 | 40.6 | 45.4 | 29.8 | 41.6 | 41.5 | 36.5 |
| 75-84 | 40.9 | 40.3 | 42.1 | 42.4 | 41.9 | 37.7 |
| **85+** | 18.5 | 14.4 | 28.1 | 15.9 | 16.6 | 25.8 |
| **Female Sex** | 58.2 | 55.9 | 64.4 | 56.2 | 60.3 | 53.5 |
| **Race and ethnicity** | | | | | | |
| Non-Hispanic White | 85.2 | 83.8 | 88.1 | 87.4 | 82.4 | 84.4 |
| Non-Hispanic Black | 10.8 | 12.0 | 8.5 | 8.7 | 13.6 | 10.4 |
| Hispanic | 1.1 | 1.1 | 1.0 | 0.9 | 1.3 | 1.3 |
| Other | 3.0 | 3.2 | 2.5 | 2.9 | 2.7 | 4.0 |
| **Medicaid dual enrollment** | 37.8 | 34.8 | 44.8 | 35.5 | 43.8 | 35.8 |
| **Year of hospitalization** |  |  |  |  |  |  |
| 2016 | 31.7 | 31.4 | 32.5 | 34.2 | 34.0 | 31.8 |
| 2017 | 34.0 | 34.0 | 33.7 | 35.0 | 31.4 | 34.7 |
| 2018 | 34.3 | 34.6 | 33.7 | 30.9 | 34.5 | 33.5 |
| *Hospital stay attributes* |  |  |  |  |  |  |
| Length of hospital stay, mean (SD), days | 5.5 (6.0) | 4.5 (4.8) | 7.2 (6.9) | 6.5 (7.2) | 4.1 (4.2) | 8.3 (10.0) |
| Length of hospital stay ≥3 days, % | 73.6 | 65.6 | 93.4 | 74.0 | 57.9 | 70.0 |
| Combined Comorbidity Score, mean (SD) | 3.4 (2.2) | 3.2 (2.1) | 3.8 (2.2) | 3.4 (2.1) | 3.6 (2.2) | 4.3 (2.2) |
| Comorbidity count ≥3, % | 59.8 | 55.4 | 67.6 | 60.2 | 63.9 | 76.1 |
| Intensive care unit use, % | 34.6 | 30.9 | 36.2 | 45.3 | 30.3 | 69.6 |
| *Post-hospital discharge outcomes within 30 days* |  |  |  |  |  |  |
| All-cause acute readmission, % | 26.1^c^ | 23.8 | 24.5 | 42.0 | 35.8 | - |
| All-cause mortality, % | 6.2^c^ | 5.5 | 6.6 | 9.4 | 6.7 | - |

*Non-SNF institutional settings include inpatient rehabilitation facilities, long term care hospitals, psychiatric hospitals

^a^Unless otherwise indicated

^b^Row percent; whereas, other percentages are column percents

^c^Denominator for percent is 178,372 excluding in-hospital deaths

**Appendix S4**: Demographic, comorbidity and health care utilization characteristics of opioid use disorder-related hospitalizations among younger (age <65 years) fee-for-service Medicare beneficiaries, 2016-18

|  |  | Hospital discharge location | | | | |
| --- | --- | --- | --- | --- | --- | --- |
| Characteristic, %^a^ | Overall | Home without home health | Skilled nursing facility (SNF) | Non-SNF institutional setting* | Home with home health | In-hospital death |
|  | N=276,874 | n=214,413 | n=24,899 | n=27,189 | n=6,599 | n= 3,774 |
| **Discharge location,** **as percent of total stays**^b^ | 100 | 77.4 | 9.0 | 9.8 | 2.4 | 1.4 |
| **Age, mean (SD), years** | 50.5 (10.4) | 49.9 (10.5) | 55.5 (8.0) | 49.9 (10.6) | 52.5 (9.5) | 53.4 (9.3) |
| 18-49 | 40.6 | 43.3 | 20.1 | 43.1 | 32.9 | 28.6 |
| 50-64 | 59.4 | 56.9 | 79.9 | 56.9 | 67.1 | 71.4 |
| **Female Sex** | 50.7 | 50.8 | 52.2 | 49.0 | 54.8 | 45.7 |
| **Race and ethnicity** | | | | | | |
| Non-Hispanic White | 76.5 | 75.8 | 79.6 | 79.7 | 73.6 | 77.5 |
| Non-Hispanic Black | 16.5 | 17.0 | 14.8 | 13.9 | 19.9 | 15.6 |
| Hispanic | 3.4 | 3.5 | 2.7 | 2.9 | 3.3 | 3.6 |
| Other | 3.6 | 3.7 | 3.0 | 3.6 | 3.2 | 3.3 |
| **Medicaid dual enrollment** | 73.2 | 72.3 | 79.2 | 74.9 | 78.8 | 65.8 |
| **Year of hospitalization** |  |  |  |  |  |  |
| 2016 | 35.5 | 35.6 | 34.4 | 35.8 | 35.8 | 36.0 |
| 2017 | 34.0 | 33.9 | 34.7 | 34.9 | 30.8 | 33.4 |
| 2018 | 30.5 | 30.5 | 30.9 | 29.3 | 33.3 | 30.6 |
| *Hospital stay attributes* |  |  |  |  |  |  |
| Length of hospital stay, mean (SD), days | 5.5 (7.0) | 5.0 (6.1) | 8.9 (10.0) | 6.3 (8.3) | 5.0 (6.1) | 8.2 (12.4) |
| Length of hospital stay ≥3 days, % | 69.8 | 67.6 | 92.9 | 68.1 | 62.3 | 66.5 |
| Combined Comorbidity Score, mean (SD) | 2.4 (2.0) | 2.2 (1.9) | 3.3 (2.2) | 2.4 (2.0) | 2.9 (2.1) | 3.5 (2.1) |
| Comorbidity count ≥3, % | 39.1 | 36.1 | 57.8 | 39.8 | 49.3 | 63.6 |
| Intensive care unit use, % | 29.1 | 25.8 | 38.6 | 40.2 | 28.2 | 77.0 |
| *Post-hospital discharge outcomes within 30 days* |  |  |  |  |  |  |
| All-cause acute readmission, % | 32.1^c^ | 29.7 | 29.7 | 50.8 | 43.8 | - |
| All-cause mortality, % | 2.4^c^ | 1.8 | 4.0 | 4.6 | 3.2 | - |

*Non-SNF institutional settings include inpatient rehabilitation facilities, long term care hospitals, psychiatric hospitals

^a^Unless otherwise indicated

^b^Row percent; whereas, other percentages are column percents

^c^Denominator for percent is 273,100 excluding in-hospital deaths
